# Contextual factors affecting the implementation of an anemia focused virtual counseling intervention for pregnant women in plains Nepal: a mixed methods process evaluation

**DOI:** 10.1186/s12889-023-16195-5

**Published:** 2023-07-06

**Authors:** Sanju Bhattarai, Samata Kumari Yadav, Bibhu Thapaliya, Santosh Giri, Basudev Bhattarai, Suprich Sapkota, Shraddha Manandhar, Abriti Arjyal, Naomi Saville, Helen Harris-Fry, Hassan Haghparast-Bidgoli, Andrew Copas, Sara Hillman, Sushil Chandra Baral, Joanna Morrison

**Affiliations:** 1 HERD International, Sainbu Awas Cr-10 Marga, Bhaisepati, Lalitpur, Nepal; 2 UCL Institute for Global Health, 30 Guilford Street, London, WC1N 1EH UK; 3 London School of Hygiene & Tropical Medicine, Keppel St, London, WC1E 7HT UK; 4 UCL Institute for Women’s Health, Rm 237C Medical School Building, 74 Huntley Street, London, WC1E 6AU UK

**Keywords:** Anemia, Nutrition, Pregnancy, Nepal, mHealth, Antenatal, Iron and folic acid, Virtual

## Abstract

**Background:**

Anemia is estimated to cause 115,000 maternal deaths each year. In Nepal, 46% of pregnant women have anemia. As part of an integrated anemia-prevention strategy, family engagement and counseling of pregnant women can increase compliance to iron folic acid tablets, but marginalized women often have lower access to these interventions. We implemented the VALID (Virtual antenatal intervention for improved diet and iron intake) randomized controlled trial to test a family-focused virtual counseling mHealth intervention designed to inclusively increase iron folic acid compliance in rural Nepal; here we report findings from our process evaluation research.

**Methods:**

We conducted semi structured interviews with 20 pregnant women who had received the intervention, eight husbands, seven mothers-in-laws and four health workers. We did four focus groups discussions with intervention implementers, 39 observations of counseling, and used routine monitoring data in our evaluation. We used inductive and deductive analysis of qualitative data, and descriptive statistics of monitoring data.

**Results:**

We were able to implement the intervention largely as planned and all participants liked the dialogical counseling approach and use of story-telling to trigger conversation. However, an unreliable and inaccessible mobile network impeded training families about how to use the mobile device, arrange the counseling time, and conduct the counseling. Women were not equally confident using mobile devices, and the need to frequently visit households to troubleshoot negated the virtual nature of the intervention for some. Women’s lack of agency restricted both their ability to speak freely and their mobility, which meant that some women were unable to move to areas with better mobile reception. It was difficult for some women to schedule the counseling, as there were competing demands on their time. Family members were difficult to engage because they were often working outside the home; the small screen made it difficult to interact, and some women were uncomfortable speaking in front of family members.

**Conclusions:**

It is important to understand gender norms, mobile access, and mobile literacy before implementing an mHealth intervention. The contextual barriers to implementation meant that we were not able to engage family members as much as we had hoped, and we were not able to minimize in-person contact with families. We recommend a flexible approach to mHealth interventions which can be responsive to local context and the situation of participants. Home visits may be more effective for those women who are most marginalized, lack confidence in using a mobile device, and where internet access is poor.

## Introduction

Anemia in pregnancy is estimated to cause around 115,000 maternal deaths per year [1, 2] and is a particular problem in low-and middle-income countries (LMICs). A systematic review to estimate the prevalence of anemia in LMICs showed that 43% of women experienced anemia during pregnancy [3]. Iron and folic acid (IFA) supplementation, deworming and nutrition counseling can prevent anemia in pregnancy [4, 5] and are widely delivered as a part of routine antenatal care. Despite this, anemia remains a persistent problem, and there is a need to develop and evaluate interventions which reach the most marginalized pregnant women in LMICs.

We developed an mHealth intervention for pregnant women and their families in rural Nepal and tested its effectiveness on uptake of IFA supplements, dietary diversity, and number of antenatal care visits through a randomized controlled trial [6]. In this paper we report the findings of our process evaluation which evaluated fidelity to the protocol and explored how contextual and design features of the intervention affected its implementation. We discuss the implications of our findings for the design of future mHealth interventions in LMICs.

### Causes of anemia in pregnancy in South Asia

Countries in South Asia are not making progress to meet the World Health Assembly target of reducing anemia among reproductive age women by 50% by 2025 [7, 8]. Poor diet is a significant driver of anemia in pregnancy. South Asia had the highest estimated prevalence of inadequate intake of iron and vitamin C for all years between 1961 and 2011 and most years for vitamin A, vitamin B12, and zinc [9].

Early pregnancy combined with short birth spacing can leave women and girls depleted of essential nutrients [10]. Infection also contributes to anemia prevalence, particularly in rural areas where many women and girls lack access to clean water, hygiene, and sanitation [11]. Poverty and gender differences in household allocation of food are also contributors to anemia in South Asia [12].

### Addressing anemia in pregnancy in Nepal

The Nepal Demographic Health Survey reported that 34% of reproductive age women and 33% of pregnant women had anemia [13]. Women living in the plains were more likely to be anemic (45%) than those living in the hills (20%) and mountain (23%) regions [13]. In Nepal, deworming, free IFA and nutrition counseling are provided from the first antenatal care visit at 16 weeks of pregnancy [14], but the most marginalized are the least likely to access these services. 75% of women in the lowest wealth quintile attended four or more ANC visits which is around 18 percentage points lower than those women in the highest wealth quintile at 92.6% [13]. A review of maternal IFA supplementation programs in Nepal found that although 91% of women took IFA supplements during pregnancy, only 71% took them for at least 90 days and only 42% for at least 180 days [15]. The proportion of pregnant women who took IFA supplements for at least 90 days was lower in the plains than in the hills (67% vs. 76%) and women in the lowest wealth quintile were less likely to take the recommended IFA dose than women in the highest quintile (67% vs. 84%) [15].

The Government of Nepal recognises that distance to health facilities and the need for accompaniment can limit access to health facilities for poor women living in remote areas [16]. Therefore, outreach clinics are run by health workers and female community health volunteers (FCHVs) to provide access to ANC. FCHVs also identify and encourage pregnant women to attend ANC and can also re-supply IFA. However, the effectiveness of FCHVs at addressing anemia in pregnancy has not been evaluated, and they are often inadequately supported to provide nutrition counseling [17, 18].

Engaging the wider family is important to ensure access to ANC, IFA, and adequate and diverse diets, particularly because the risk of anemia in pregnancy varies by family circumstances [19, 20]. Providing face-to-face home-based counseling by a trained health worker may not be possible among scattered populations but mHealth interventions using virtual counseling may offer a more feasible counseling delivery mechanism.

### mHealth for improved maternal health

mHealth interventions are often suggested as a way to improve access to health care in remote areas, where there are shortages of skilled teams of health workers, and there are substantial opportunity costs to accessing care [21]. Most research on mHealth interventions in pregnancy has occurred in high income countries [22, 23], but there are some examples from LMICs. For example, mHealth interventions have been used to: educate women about warning signs in pregnancy through text messaging [24]; to provide remote supervision and support to rural health workers [25]; and to remind pregnant women to attend ANC [26]. mHealth interventions during pregnancy in LMICs have used text or voice messages [24–35]or verbal communication [25, 28, 29, 35–37]. The COVID-19 pandemic further stimulated the use of mHealth interventions [38–40].

### The VALID mHealth virtual counseling intervention

We implemented virtual counseling (VC) through a Zoom communications platform on mobile devices from 23rd January to 6th May 2022 in rural plains Nepal when COVID-19 infection rates were low. We provided two VC sessions to married pregnant women aged 13–49. The first session was given between 12 and 28 weeks of pregnancy. We aimed to provide the second VC at least 14 days from the first VC. Informed by formative research, we developed a dialogical counseling guide for use by trained counselors to virtually engage pregnant women and their family members in discussions about anemia prevention. The guide was informed by Freire’s empowerment education model which proposes a ‘listening-dialogue–action’ approach [41]. First, the counselors listened to the experiences of women and family members about the pregnancy generally. The counselors then read a story which referred to common problems raised in formative research. The story acted as a ‘trigger’ for discussion [42]. Next, the counselors developed a dialogue with participants about the factors affecting access to ANC, the factors affecting IFA consumption and consumption of a diverse, iron rich diet. This could include counselors posing problems and discussing them to develop a shared understanding about the potential causes of anemia specific to that woman in her family context. Lastly, counselors and participants developed an action plan to address the issues discussed. This dialogical approach has been more commonly used in community mobilisation interventions but given our knowledge about the gendered and household drivers of anemia in this context, and safety concerns of a group-based approach during the pandemic, we sought to test its effectiveness as a family-based counseling approach [19].

In the second VC, the counselors used a similar approach which started by discussing what had been successful and what had been challenging while implementing the action plan. Then, the counselors read another story which covered different issues from the first story. The story was then used to trigger discussion and development of a revised action plan.

The intervention was implemented by 10 female counselors who were qualified as auxiliary nurse midwives, or graduates with more than 4 years’ experience implementing health and nutrition interventions in the community. Most of them were from the local area and all spoke Awadhi and Nepali languages. Counselors received six days of training on anemia and nutrition during pregnancy, interpersonal communication, and using the guide to facilitate VC. They learned how to use mobile devices and Zoom, and how to teach pregnant women over the phone about their device. We used role plays of how to use the counseling guideline based on examples from formative research. Each counselor conducted eight role plays during training and one with community women who were not pregnant but had had children within the last year.

### Implementation of the intervention

We identified pregnant women through a community surveillance system [6]. Researchers identified pregnant women in the study areas with help from incentivised FCHVs. Pregnant women were then visited by a researcher and given a mobile device with Zoom and Whatsapp applications already installed. The mobile device contained a sim card with 399 Nepalese rupees data (3 USD). Field researchers taught women and their families how to use the device and provided a paper copy of screenshots of step-by-step instructions. Counselors then called each pregnant woman to schedule the 60–90-min VC over Zoom. After the VC, the action plan was audio recorded in the preferred language and sent to the device through Whatsapp. A generic educational video message on nutrition and ways to prevent COVID-19 was also sent to women through Whatsapp. Counselors implemented the VC from a call center in Kapilvastu and were supervised by an intervention coordinator who listened to the first VC done by each counselor, off-screen and in real time. He gave feedback and was in the call center to listen, troubleshoot and identify counselors who required additional training.

## Methods

### Study setting

We did this research in the southern part of Kapilvastu district, Lumbini Province, in the plains of central Nepal. Kapilvastu has a population of around 508,500, a mean household size of 5.5 members [43] and is in the second least developed category of districts in Nepal with a Human Development Index of 0.452 [44]. 85% of households use a tube well for drinking water, and 74% of households have no toilet [43]. Most of the population are engaged in farming, and just under half of women are literate, compared with 65% of men [43]. Lumbini Province had the largest number of outgoing international migrants in Nepal according to 2011 census [45]. This is of relevance for our research as migrants often communicate through mobile devices with their family members, and therefore households may be more familiar with virtual platforms than in areas with fewer migrants.

Most of the population are of plains (Madhesi) ethnicity, and 68% of the population speak Awadhi language [46]. 13% of the population are from disadvantaged low caste (Dalit) groups, 17% are from indigenous groups (Janjati) and 18% are Muslim [47]. Discriminatory gender-based practices and unequal power relations affect the wellbeing of women, and are common in Madheshi and Muslim communities [48]^.^ Lowest median ages of marriage for girls are found in Lumbini province [49] and women's movement outside of the home is strictly controlled. Research has found that defying these norms is used to justify intimate partner violence [50].

The objectives of our mixed methods process evaluation were to:

- 1. Evaluate the extent to which the intervention was implemented according to plans (fidelity to plans).
- 2. Analyze how context interacted with the intervention
- 3. Develop hypotheses about how the intervention worked or failed to work.

In this paper, we describe the methods and present the findings related to the first two objectives. Our analysis of the third objective will be written about in a forthcoming publication. We collected data from seven different data sources: trial surveys, call logs, routine monitoring data, direct observations, semi-structured interviews (SSIs), and focus group discussions (FGDs) (Table 1).

**Table 1:** Data sources

Data source	Process evaluation component
VALID trial surveys of 316 pregnant women at baseline, and 271 at endline [6]	Context and mechanisms
Call log maintained by counselors to document number of calls and reasons for calling pregnant women during the intervention	Implementation
VC monitoring forms filled by counselors after each VC documenting participation of family members, description of the action plan, implementation of the action plan, factors affecting the VC and participation in the VC	Implementation and context
Daily log maintained by the Intervention Coordinator documenting problems encountered in delivering the intervention and how they were managed	Implementation
Qualitative observations of VC to assess fidelity to the protocol	Implementation and context
SSIs with pregnant women (20), their mothers-in-laws (8), husbands (7) and health workers (4) to explore their experience of the intervention, facilitators and barriers to receiving the intervention	Implementation, context, and mechanisms
2 FGDs with counselors and 2 FGDs with field researchers to explore the factors affecting implementation, and explore counselor definitions of a ‘good’ counseling session	Implementation and context

### Quantitative data

Quantitative data from trial surveys and routine implementation monitoring data were used. Trial surveys captured data on sociodemographic status of 319 pregnant women enrolled in the trial; their access to and familiarity with mobile phones, smart phones, and using the internet; participation of family members in the VC; and whether an action plan was developed after the VC. Trained researchers enrolled and interviewed 316 pregnant women at median 18.5 weeks (range 7 to 29 weeks) gestation, and with 271 participants at endline (29 to 97 days after enrolment). Methods for the trial survey are described in detail elsewhere [6]. Routine monitoring data from call logs maintained by counselors were used to describe how many calls were made and the reasons for these calls.

### Qualitative observations

We conducted semi-structured observations of VCs using VC monitoring forms. All VCs were recorded, and we observed a purposive sample of 15% of VCs (*n* = 39). We theorised that the skills of the counselor, and the parity of the woman might have influenced their experience of the intervention, and therefore we purposively sampled two VCs conducted by each counselor with different women and ensured representation of women who were having their first pregnancy. Around half of the observations were of the first counseling session and half were of the second session. We present the characteristics of those women in Table 2.

**Table 2:** Sociodemographic information of women whose VCs were observed and of women whose VC monitoring forms were analyzed

	**Observed First VC (*N* = 19)** **n(%)**	**Observed Second VC (*N* = 20)** **n(%)**	**VC monitoring forms analysed** (*N* = 26) **n(%)**	**Total population** **(*N* = 319)** **n(%)**
Ethnicity^a^				
Terai/Madhesi Dalit	**6 (31.6)**	**4 (20.0)**	**5 (19.2)**	**55 (17.2)**
Terai/Madhesi Janajati	**0 (0.0)**	**1 (5.0)**	**0 (0.0)**	**5 (1.6)**
Terai/Madhesi Muslim	**3 (15.8)**	**6 (30.0)**	**10 (38.5)**	**92 (28.8)**
Terai/Madhesi Other castes	**9 (47.4)**	**8 (40.0)**	**11 (42.3)**	**159 (49.8)**
Terai/Madhesi Brahmin/Chettri	**1 (5.3)**	**0 (0.0)**	**0 (0.0)**	**5 (1.6)**
Hill Brahmin/Chettri	**0 (0.0)**	**1 (5.0)**	**0 (0.0)**	**3 (0.9)**
Parity (number of live births)				
Nulliparity (0)	**7 (36.8)**	**9 (45.0)**	**7 (26.9)**	**74 (23.2)**
Low multiparity (1–3)	**12 (63.2)**	**6 (30.0)**	**13 (50.0)**	**201 (63.0)**
Grand multipara (4–8)	**0 (0.0)**	**5 (25.0)**	**6 (23.1)**	**44 (13.8)**
Education				
No schooling	**5 (26.3)**	**8 (40.0)**	**11 (42.3)**	**112 (35.1)**
Primary (1–5 grade)	**6 (31.6)**	**5 (25.0)**	**8 (30.8)**	**105 (32.9)**
Upper Primary (6–8 grade)	**3 (15.8)**	**2 (10.0)**	**2 (7.7)**	**44 (13.8)**
Secondary (9–10 grades)	**3 (15.8)**	**3 (15.0)**	**2 (7.7)**	**42 (13.2)**
Higher Secondary (11 -12 & Bachelors)	**2 (10.5)**	**2 (10.0)**	**3 (11.5)**	**16 (5.0)**

^a^based on classification in [51]

Observation forms were used by a process evaluation officer who collected data on the five key components of the intervention detailed in Table 3. From the remaining unobserved VCs, we randomly sampled 20% (*n* = 52 forms from 26 women) VC monitoring forms that were filled in after each VC by counselors (Table 2). These contained closed questions about participation of family members, description of the action plan, implementation of the action plan, and open questions about factors affecting the VC and participation in the VC.

**Table 3:** Fidelity to plans and adjustments

Key intervention components	Fidelity (low, medium, high)	Reasons for low or medium fidelity and any adjustments made
*Before the VC*		
Researcher visits house once to deliver the mobile device with 4–7 GB (3 USD) data & train family to use	Low	Researchers made many visits to houses to train family on tablet use. This was often because they couldn’t demonstrate while there were internet problems In 3 cases the data provided had been used before the first VC, and additional data was provided All women were given additional data so they could receive the action plan
Pregnant woman takes ownership of device	Medium	A few (*n* = 3) women refused to keep the mobile device because they feared it would get damaged so neighbors or an FCHV kept it for them
NA call once to schedule the VC, and once to confirm receipt of zoom link and remind to charge the device (maximum 5 calls for each VC in 48 h)	Low	An average of 4 calls were made to arrange the VC, but this ranged from 1 to 72 calls Lack of familiarity with zoom and mobile devices, internet problems, and difficulties scheduling increased the number of calls
*During the VC*		
Duration around 60–90 min	Medium	First counseling took an average of 62 min (Range 43–101 min) and the second VC took an average of 53 min (Range 35–89 min) VC was frequently interrupted by internet problems, and this prolonged the VC and interrupted the ‘flow’ of the discussion
Story with picture cards shown on screen to trigger discussion	Medium	The story was used and felt to be effective to trigger discussion Sometimes the counselor forgot to change the pictures as she was telling the story, and the screen share was not turned off after the story was complete
Dialogical approach, led by problems mentioned by women and her family	Medium	The discussions were not always dialogical. Counselors encouraged women and family members to share their concerns but found it difficult to help them find solutions when they were not linked with anemia Discussions often ended in counselor’s giving a lot of information to the woman and her family
Focus on family engagement when finding solutions to problems	Low	Family members participated in nearly 60% of the VCs. However, their interaction was minimal, and some were unwilling or unable to appear on camera as it was difficult to share the small screen Many family members did not participate in the whole counselling session, leaving before the end, or participating sporadically When women had to receive calls outside their homes (because of internet problems) it was difficult for family members to participate
Make a family action plan at the end of the VC to address barriers to preventing anemia	Medium	Most women made action plans (102 first VC and 96 in second VC) but in most cases plans focused on what action would be taken (ie drink more water) and not on how to take action (ie husband agrees to remind woman to drink more water)
At the second VC, review the action plan	Medium	Action plans were reviewed but in some cases the counselor did not discuss the challenges of implementing the action plan and how to resolve these
*After the VC*		
Action plan and key messages sent to women	High	Action plans and key messages were sent as audio or text files to almost all women. One woman was worried that receiving messages on the device would ‘create trouble at home’, and she did not receive messages counselor’s called to confirm that action plans were received

### Semi-structured interviews and focus group discussions

For SSIs, we purposively sampled women who had received the VC, and husbands and mothers-in-law of women who had received the VC (Table 4). No one declined to participate. We sought maximum variation in sampling and therefore sampled women with none and some schooling; primigravida and multigravida, and women from different ethnic groups. Husbands and mothers-in-law were sampled based on their participation or non-participation in the VC and were not from the same households as women or other family members who were interviewed. We conducted 20 SSIs with women, seven SSIs with mothers-in-law, and eight SSIs with husbands using topic guides to explore their experience of the VC and explore barriers and facilitators to participating in the VC. We also asked them how the VC could be improved. In addition, we conveniently sampled four health workers who provided antenatal care in the intervention areas to explore acceptability of the intervention. We showed them a pilot VC and then interviewed them using a topic guide to explore the feasibility of the intervention and explore how context may have affected implementation.

**Table 4:** Characteristics of qualitative sample who received the intervention

Characteristics	SSI with pregnant women (20)	SSI with mothers-in-law (7)	SSI with husbands (8)
VC participation			
Participated	20	4	4
Not participated	-	3	4
Education			
No schooling	5		
Primary (1–5)	5		
Upper Primary (6–8)	6		
Secondary (9–10)	4		
Ethnicity^a^			
Terai/Madhesi Dalit	2	1	4
Terai/Madhesi Janajati	1	-	-
Terai/Madhesi Muslim	4	2	-
Terai/Madhesi Other castes	12	4	4
Hill Brahmin/Chettri	1	-	-
Parity (number of live births)			
Nulliparity (0)	5		
Low multiparity (1–3)	12		
Grand multipara (4–8)	3		
Access to a smart phone			
Yes	9		
No	11		
Experience with using the internet on the phone			
Very experienced	5		
A bit experienced	7		
No experience	8		

^a^based on classification in(51)

We conducted two FGDs with all counselors, and two FGDs with all field researchers using topic guides. Topic guides explored implementation issues identified through preliminary analysis of observation data and monitoring forms and explored how context had affected the implementation of the intervention. In addition, we asked counselors to describe a good VC, how they encouraged women and family members to participate and how the VC could be improved.

### Data management

All participants gave informed verbal or written informed consent to participate. If the participant was under the age of 18 we also took informed consent from their legal guardian. Where participants were illiterate, we explained the study to them (and their guardians if they were under the age of 18) and obtained verbal consent to participate in accordance with Nepal Health Research Council approved ethical procedures. We took additional informed consent from the women in the pilot VC to share her counseling session with health workers in order to facilitate discussions. Data were collected in Awadhi or Nepali (depending on the preference of the participant) by two bilingual trained qualitative researchers who were working as process evaluation officers. SSIs and FGDs were audio recorded and transcribed verbatim in Nepali and translated into English for analysis. Observation reports, monitoring forms, and trial surveys were in Nepali. Survey and call log data were collected using a structured Nepali form in Commcare.

### Data analysis

Initially, we analyzed data sources separately. Survey and call log data were downloaded from the CommCare server and analyzed to calculate frequencies and proportions using Stata by SG and NS. FGD and SSI data from women and family members were analyzed by BT, SY, SB and AA. SY and BT separately read and made notes on three SSI transcripts (1 woman and 2 family members). They then discussed and generated codes which were used to code the remaining data in Excel. Each row represented an SSI or FGD and each column a code.

One observation and one monitoring form were discussed collectively by BT, JM, NS, and SM to create codes under the headings 1) factors affecting implementation, and 2) factors affecting action. These codes were then used by BT and SY to analyze all observation and monitoring forms in excel.

JM read data from counselors, health workers and field researchers and created codes under the topic guide headings ‘what makes a good VC’, ‘participation in the VC’, ‘factors affecting implementation’, and ‘areas for improvement’, and compared these findings with the data from women, husbands, and mothers-in-law through discussion with SY and BT.

Four descriptive reports of each data source were written by BT, SY, and SG. To integrate analysis across the data sources these were read, discussed, and adjusted with JM and SB, and then the main interpretations relating to our research objectives were presented to the wider team for discussion and validation.

## Results

**Fig. 1:**
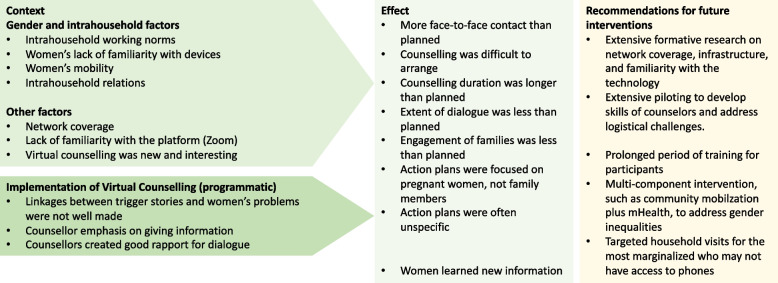
Context and implementation factors affecting the response to the intervention

We first present findings about the contextual barriers and enablers which affected the implementation and response to the intervention such as gender, intrahousehold factors, network coverage, lack of familiarity with zoom. Thereafter we present the programmatic factors such as counselor emphasis on giving information and poor linkage between the trigger stories and women’s problems. An overview of these factors is presented in Fig. 1.

### Contextual factors affecting the implementation of the intervention *Poor internet reception*

There was unreliable internet reception in most of the district, and so it took longer than planned to teach families how to use the mobile device and Zoom link: *“It takes 1–2 h to instruct one person. It is a bit of a problem when there is no network connection while teaching them to use the [mobile device]” (FGD field researchers).* For some women, field researchers were able to give families a sim card from a provider that gave better coverage. In areas bordering India it was not possible to do the VC, but this only affected a few women who received audio counseling over the phone: *“How could we do a video call when neither mobile company is working? There were problems in the areas near the Indian border. We tried … but only phone worked, video call didn’t work*” *(FGD field researchers).*

When there was poor network coverage, the VC was often interrupted which made the session longer than planned and difficult to conduct. Women felt understandably impatient when this happened. SSIs and data from call logs indicate that women from marginalized groups (Muslim and Dalit) were more likely than other women to have to move around searching for better reception: *“The internet was disconnected two to three times during the counseling session. I faced this problem a lot and I had to go to outside in front of the house so many times” (Dalit pregnant woman with no schooling).* Some women had to go to another person’s house for the VC: *“There was no network during the counseling, so I went to my neighbor’s house” (Dalit pregnant woman with primary education).* Occasionally, the VC had to be rescheduled because of power cuts and mobile devices not being sufficiently charged: “*The call disconnected as my mobile got switched off. I called the person who gave me the mobile. She told me to charge the mobile before the counseling and asked for my next available time to continue the counseling” (Dalit pregnant woman with primary education).*

**Fig. 2:**
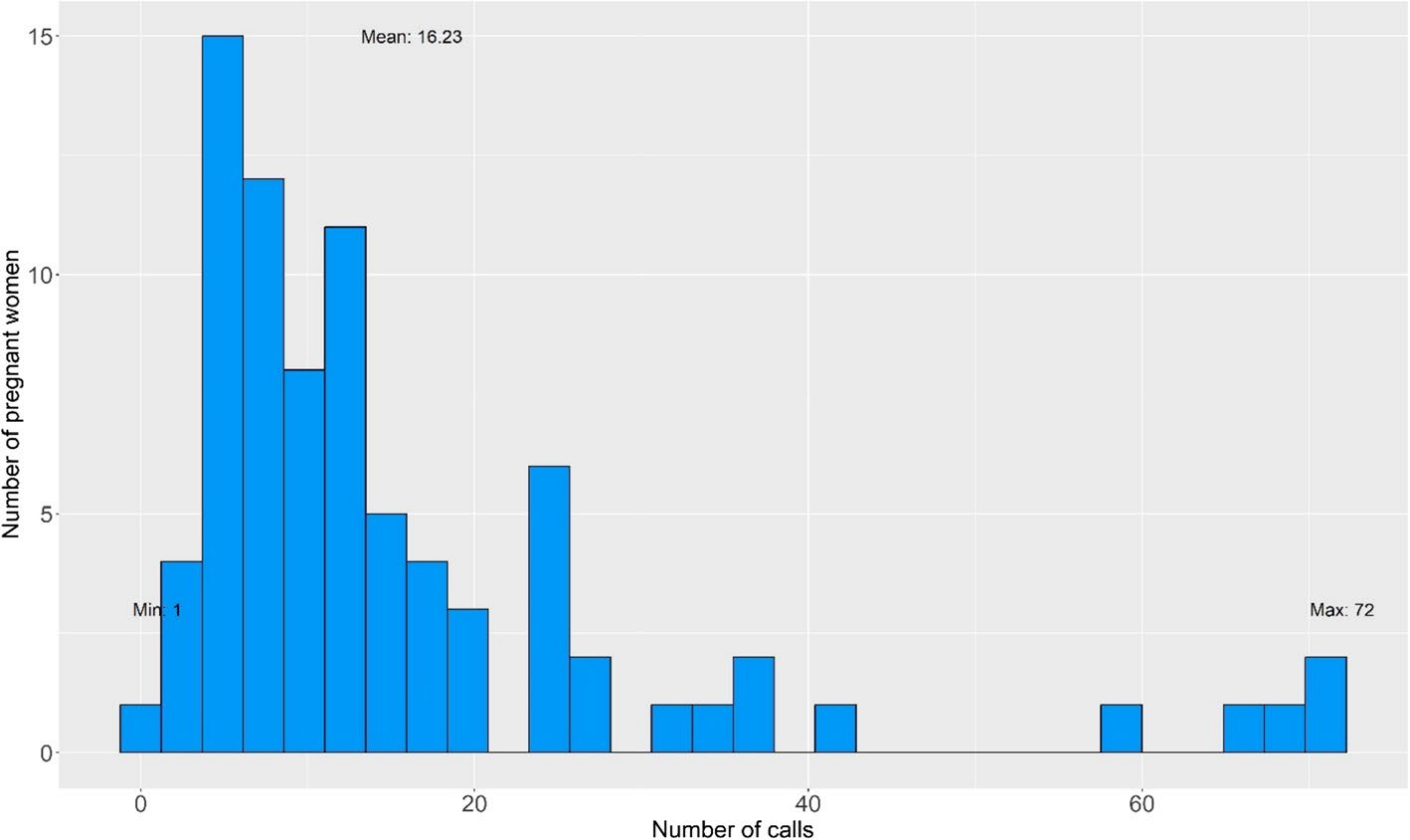
Number of calls to the 158 women enrolled in the intervention from call log data

We expected counselors to make up to five calls before a VC, but an average of 16 calls were made to schedule and conduct the VC as shown in Fig. 2. 35% (851/2404) of the calls that the counselor made were to provide technical support to use the mobile device and open Zoom link (Table 5).

**Table 5:** Reasons for calling women

Reasons	Frequency (%) *N* = 2404
Schedule time for 1st counseling	654 (27.2)
Schedule time for 2nd VC	899 (37.4)
To enquire about the Zoom link	244 (10.1)
To start the VC	218 (9.1)
Mobile device handover, collection, and replacement	154 (6.4)
To enquire about data pack/turn data on–off	144 (6)
Follow-up enquiry^a^	63 (2.6)
Technical enquiry^b^	25 (1)
Other reasons^c^	3 (0.1)
Average calls (Min—Max)	16.24 (1—72)

^a^*Enquiries about the status of the pregnant women, reminders about action plan, completion of interrupted sessions*

^*b*^*enquiries about network connection issues, tablet usage, and keeping the tablet charged*

^*c*^*returning a woman’s call or calling on request later*

### Gender norms

#### Gendered working norms

Initially, many women said that scheduling would not be a problem as they were usually around the house, but they were often busy with household or farm work: *“Initially, I told the counselor she could call me any day and any time, but when she called me at 4 pm the next day, I was washing dishes, so it had to be cancelled. We were able to talk the next day”* (Madheshi pregnant woman with primary education). Women often had to prioritize household tasks during the VC which made them distracted. This also lengthened VC because the counselor paused to fit around women’s needs. Many women asked the counselor to complete the VC quickly so they could resume their work, and this affected the interactive nature of the VC: *“Although they give us their time, they spoke to us with people around them and worked while we are speaking”* (FGD counselor).

Intrahousehold and gender roles resulted in women often being alone at home during the day, which meant it was difficult for other family members to participate. Men were particularly difficult to engage: *“Everybody goes to work between 10 am to 10 pm. The men in their home are usually out. Only women are there, and even then, mothers-in-law are frequently out in the field working and there’s only the pregnant woman in the house”* (FGD counselor*)*. Consistent with this, the monitoring forms showed that only 59% (*n* = 75) of VCs had a family member participating, and of these VCs only 28% (*n* = 20) were husbands (Table 6).

**Table 6:** Participation of family members in VC

Family members	Participation *N* = 75 (%)
Husband	20 (27.8)
Mother-in-law	33 (38.8)
Other men	12 (13.3)
Other women	46 (43.5)

#### Gendered access to phones

Before the intervention 47% (*n* = 59) of women in the trial said they were familiar with smart mobile devices or had access to these but during the trial we found that they often needed others’ help to use them. There were no clear patterns about what affected phone literacy. One woman with access to a smartphone said: “*My husband dials the number for me whenever I have to talk to somebody. Nowadays, even small children know how to use a mobile, but I don’t know…. The researchers oriented me, but I could not learn*” (Muslim pregnant woman with primary education). Women’s lack of ownership and limited access to mobile phones affected their confidence and familiarity with the device, and so affected their participation in the VC. Field researchers had to visit households many times to teach the woman and family how to use the device: *“Researchers visited 2–3 times and opened the link for me. I don’t know how to use mobile*” (Dalit pregnant woman with primary education). After the device was given to the family, we tried to conduct all communication over the phone or virtually, but this was usually not possible. Most participants were unfamiliar with Zoom which was problematic*: “(some women) asked us to conduct counseling physically or through a phone call. They said they found it difficult to open the Zoom link”* (FGD counselor*).*

Women often relied on family members to help them during the VC: “*My daughter set up the mobile device, opened the Zoom link, and gave it to me. And I listened and participated in the virtual counseling. After the session was finished, (my daughter) turned it off for me* (Madheshi pregnant woman with no schooling). When family members were not available, women had to seek help from neighbors to join the virtual session: *“My husband and brother-in-law helped me to open the mobile device and Zoom link as I couldn’t do it. When they were not available, I used to call other people in the village (to help)”* (Muslim pregnant woman with no schooling).

The contact information for pregnant woman was often the husband’s number, and he was usually outside the house working when the counselor called to schedule or conduct a VC: *“Sometimes women don’t have their [personal mobile] and their husband picks up and says: ‘I’m out of the house at the moment and will only reach home later’. That was a problem”* (FGD counselor). Counselors also said that some women who had their own phone did not feel comfortable to disclose their number.

#### Intra-household hierarchy and women’s agency

Counselors were pleasantly surprised that most women were able to talk openly to them over a virtual platform: “*In the beginning, it was hard to believe that the women of Kapilvastu would open-up to strangers. I was happy to see them willing to share their pregnancy issues. Some of them even contacted me after the session to ask if we were talking again*” (FGD counselor). In contrast, some women were reluctant to speak in front of other family members, especially their mothers-in-law: “*Often, pregnant women deliberately did not invite their mother-in-law to join. When their mothers-in-law joined, the pregnant women would not speak much in front of them*” (FGD counselor). Counselors found this problematic, as they knew the intervention targeted the family, but they observed the woman’s wishes and conducted the counseling alone if she preferred: *“We were told to involve family members in the counseling, however many pregnant women didn’t want to talk about their problems in front of their family members…They didn’t feel comfortable…They felt uneasy because if they share their difficulties, their family members might say something bad later….They didn’t let other family members stay. They said, “I’ll do it alone”. If the family members stayed, then they didn’t share their opinions…When they were alone, they said: ‘Family members don’t help me…they don’t understand these things. They don’t allow me to go outside.’ They were afraid of their in-laws”* (FGD counselor). On the other hand, counselors said that women often felt free to speak in front of their husbands. One health worker and an FCHV felt that whoever was the ‘guardian’ of the woman should participate in the counseling, but also acknowledged that counseling could create family problems.

Often, women’s VC participation depended on her husbands’ permission. Whilst most gave permission, three women were not allowed to participate. Some husbands did not directly refuse permission, but were unsupportive, using the data, refusing to return the device, or breaking the charger: *“They said: ‘why should we return it to them?’ I explained it and they returned it. Some of them broke the charger and threw it away, some didn’t want to return the charger”* (FGD Field researcher). Call log data show that one Muslim woman asked us not to send her action plan on the phone as this would cause trouble at home, and three women were afraid the device would be damaged and so it was kept in a neighbor’s house.

#### Women’s mobility

Some families, especially Madheshi Muslims did not allow young newly married women to go outside their home, even to the terrace or compound of the house. This meant that when their mobile network reception was not good, it was difficult for them to move to an area with better reception. Counselors told us about some examples: *“There was no network downstairs. So, she needed to go to the terrace…she said, ‘I can’t go on the terrace.’…Daughters-in-law are not allowed to go to the terrace… in my case, she was allowed to go to the terrace but not to the Aagan [courtyard outside house]…women can’t go to the terrace, they can’t go outside the gate. They need to stay inside the room”* (FGD counselor). Those women who were able to go outside their house for better mobile reception sometimes felt uneasy having the discussion about their pregnancy in a neighbor’s house: *“In one case, there was no network in the house, and she didn’t want to go to another person’s house.”* (FGD counselor*).* Women were also sometimes disturbed by other people in or around the house who were not actively participating in the VC but were curious or wanted to listen.

### The VC was new and interesting

Most women were enthusiastic about the VC, and said they learned useful and new information: “*I already knew about eating good food and going for ANC check-ups. But I found some information such as avoiding excessive tea prevents acid reflux during pregnancy more useful. (The counselor) explained properly why such practices are important”* (Muslim pregnant woman with no schooling). Women particularly enjoyed listening to and discussing the stories: *“(The* counselor*) told me the story of Kalawati. I shared all the problems that I faced. I thoroughly enjoyed the session and realized that if this information had been provided in my last pregnancy, I would not have needed a blood transfusion”* (Hill/Chettri pregnant woman with upper primary education).

Health workers who reviewed sessions said that they liked the style of the discussion and felt it was effective in getting the pregnant woman to talk about her concerns and learn new information. One said: “*I think the trial has trained counselors, or maybe they have become good counselors with practice? They know how to talk and make conversation with the women. The women of our village are usually very reluctant to talk to people outside their community”* (SSI Health post in-charge).

### Programmatic factors affecting the implementation of the intervention

#### Disjointed dialogue

Counselors shared pictures on their screen while discussing the story. They sometimes forgot to stop screen share which affected the dialogue after the story. The transition from discussing issues in the story with the woman’s own concerns was disjointed in these cases. In a few VCs, the woman’s shawl partially blocked the screen of the mobile device during storytelling and counselor did not ask women to remove it.

We had planned for a dialogical approach where the counselor would focus on issues triggered by the story that were identified by the woman and her family, but most counselors focused on providing as much information as possible to the women, even if it was unnecessary. For example, in one VC we observed a woman being urged to go for ANC when she had already been and had stated an intention to attend subsequent visits. Counselors were concerned about the VC taking too long, and their focus on information giving meant that there were fewer opportunities for discussion. In an FGD, a counselor discussed her main role as increasing knowledge: “*It’s our job to make (women) in the marginalized community aware and if they don’t have [knowledge] about something, we have messages sent to their phone which contains a lot of useful information” (FGD* counselor*).* In a few cases, counselors felt that the woman was not discussing her concerns because she didn’t want to prolong the discussion: *“(some women) listen carefully at first but as the time passes, they get irritated and don’t speak much. This might be because they think that we will take more of their [time] if they speak…They say, they don’t have any problem…. They say yes to everything and claim that they don’t have any problem and it seemed like they were irritated”* (FGD counselor). This was particularly the case for women who were busy with harvesting and/or doing daily wage labour: “One said: *“Do it in half an hour. I have work to do. I need to cut the mustard”* (FGD counselor).

### Individual focus

In addition to the difficulties in scheduling and engaging family members who were at work, observation data showed that it was difficult to engage with and make visual contact with more than one participant on a small screen. Often it was difficult to hear the counselor/participant if the screen was shared between several people which meant that family members would not be engaged throughout the VC.

In addition to the difficulties with engaging family members, we also observed that counselors focused on the pregnant woman as responsible to address problems, as opposed to encouraging a shared responsibility amongst family members. This occurred even in VCs where the family members were present. This meant that action plans were often individually focused, and rarely mentioned other household members. For example, a woman experiencing weakness was listed as the responsible person to take agreed actions of drinking adequate water, consuming nutritious food, and visiting the health facility if the situation did not improve. Action plans were often unspecific. For example, an action of ‘go for ANC’ was written instead of identifying how the action would be achieved (e.g. husband to accompany or mother-in-law to support pregnant woman to ANC). This resulted in challenges in reviewing and building on the action plan in the second VC.

## Discussion

Our research analyzed fidelity to the intervention protocol and the factors affecting the implementation of an mHealth VC intervention to pregnant women in their homes in rural plains Nepal. Our results provide lessons which are generalizable to other contexts (Fig. 1). For example, the formative work and careful intervention design helped ensure that the intervention resonated with participants. The flexibility and efforts of counselors and field researchers to ensure that women received the intervention on their terms were also positive aspects of implementation, and the intervention was largely implemented according to protocol. Implementation challenges resulted from poor internet coverage, a lack of familiarity with mobile devices and unfamiliarity with Zoom. These challenges are common in LMICs [52–55]. We underestimated the amount of support families needed with mobile devices which resulted in the intervention not being as virtual as we had planned. We were not able to consistently engage family members as planned, and gender norms and intrahousehold factors also sometimes prevented optimal participation. These findings have implications for future mHealth interventions in Nepal and in similar LMICs seeking to engage hard-to-reach pregnant women and families, and we discuss these below.

### Mobile device literacy

mHealth interventions have shown promise in addressing health needs of women in rural or remote areas [56], but we found that we had to make many visits to the household to train women and families, and to troubleshoot, which demonstrated that this intervention would not be feasible in emergency situations, or with hard-to-reach populations where multiple contacts would not be possible. Low competency of users has also been identified as an implementation challenge in other mHealth interventions in LMICs, and authors have indicated the need for a comprehensive training approach for participants [21, 52, 53, 57]. Poor access to mobile devices meant that participants were unfamiliar with the technology and required more support than was planned [53].

Using a platform which women and families were familiar with – such as IMO or WhatsApp – may have improved the implementation of the intervention, particularly as the utilisation of features (such as screen sharing) which were not available in these platforms were not particularly effective in our intervention. Instead of screen-sharing, a set of picture cards could be delivered to families with the device. Formative research should explore which platforms women and families are comfortable with and be responsive to this, explicitly checking familiarity through demonstration. Our formative research showed that many women owned and were familiar with smart mobile devices, but we found in practice that many women did not use mobile devices independently and they were not comfortable using mobile devices without the support of family members.

### Network coverage

Lack of familiarity with mobile devices might have been because the network coverage was so poor that they were infrequently used for online apps in this context. Whilst internet coverage existed throughout most of the intervention area, and we were able to deliver the VC intervention to most women, it was not reliable enough to deliver it equally to all women. Poor internet connectivity has also been an implementation challenge in other LMIC mHealth research [22]. Interventions in similar LMIC settings would benefit from formative research about mobile coverage which considers the reliability of the network, and there is a need to strengthen mobile infrastructure and electricity supply in remote areas of LMICs for mHealth to fulfill its’ potential [21, 54, 55].

### Gender norms

mHealth interventions can reinforce gender divisions strengthening men’s dominance as gatekeepers of information and mobile technology [58, 59], and we found that while most husbands gave permission for pregnant women to participate and many families supported women to receive the intervention, there were gender barriers to women’s participation. A review of mHealth interventions in LMICs found that few interventions were designed with a gender sensitive lens, and could reinforce the digital divide between men and women [60]. Globally, mHealth interventions to improve women’s health need to acknowledge and address gender inequalities, and may require community engagement as well as mHealth, or an explicit strategy to address inequalities through mHealth. For example, in Bangladesh and Afghanistan an mHealth intervention which engaged men resulted in joint decision making for the health and wellbeing of women and children [61, 62].

### Family engagement

Counselors worked with women to find a convenient time for the VC, but this often meant that we were unable to uniformly engage family members in the counseling. Sometimes women preferred not to engage family members and felt more comfortable discussing their pregnancy with counselors. Given the contextual challenges of intrahousehold hierarchies and differences in working hours of women and family members we note that family engagement may not have been an optimal intervention design. Household visits, group-based interventions, or couples counseling may have better engaged families in addressing anemia. Formative research and a prolonged period of piloting is recommended in similar LMIC settings.

## Limitations

Maximum variation sampling for qualitative data prevented comparison of findings across women with different numbers of children, different ethnic groups, and different levels of socioeconomic status. Yet our approach allowed us to identify contextual and implementation factors which were common across most groups and we were sometimes able to identify differences between marginalized and less marginalized participants. The short time frame for training and implementing the intervention affected our ability to support and supervise data collection and meant we were unable to integrate learning from process data into iterative development of intervention and implementation methods. There may have been an underestimation in the number of calls made to schedule the VC and support women. Focus group discussion data suggest that sometimes so many calls were made that counselors did not log them. Our conservative estimates of call logs already indicate a high number of calls, so we feel this is not a major limitation.

## Conclusion

mHealth interventions have potential to resolve some of the difficulties of providing health care in remote areas, but we found that contextual readiness needs to be thoroughly evaluated before intervention implementation. Where necessary, network and population capacity to use devices should be improved before testing the effect of a virtual mHealth intervention, and/or extensive field-based support should be integrated into intervention design. Multicomponent interventions are recommended in contexts of intrahousehold and gender inequalities to address the barriers to use of mobile devices for women, and a flexible approach is recommended whereby an access assessment could reveal those who would benefit most from a home visit instead of a VC intervention. Whilst health workers and many women were positive about the VC, hard-to-reach women were more likely to experience challenges in access than other women and therefore a targeted home visit approach may be preferable to a VC intervention in similar contexts.

## Data Availability

The datasets generated and/or analyzed during the current study are not publicly available because the data relate to a trial (ISRCTN 17842200) which has not yet been published. After the trial paper is published these data are available from the corresponding author on reasonable request.

## Abbreviations

ANC: Antenatal care
FCHV: Female community health volunteers
FGD: Focus group discussion
IFA: Iron and folic acid
LMIC: Low- and middle-income countries
SSI: Semi structured interview
VC: Virtual counseling
VALID: Virtual antenatal intervention for improved diet and iron intake
WHO: World Health Organization
